# Pilot Evaluation of Safe First Steps: Implementing Trauma-Informed Practices in a Hispanic School Community

**DOI:** 10.1007/s40653-025-00794-y

**Published:** 2025-11-20

**Authors:** Athena B. Thai, Lynda Gibson

**Affiliations:** 1 Department of Psychology, Rutgers University, New Brunswick, NJ USA; 2 Department of Psychology, University of Illinois Urbana-Champaign, Champaign, IL USA

**Keywords:** Adolescent, Assessment/Evaluation, Children, Program evaluation, School, Trauma

## Abstract

Minority youth are disproportionately exposed to traumatic experiences but face barriers in accessing treatment. The Substance Abuse and Mental Health Services Administration (SAMHSA) has identified six key principles for creating a trauma-informed environment, but these principles have been inconsistently implemented in schools serving diverse communities. The current study is a pilot evaluation of Safe First Steps, an initiative to implement trauma-informed practices based on SAMHSA’s key principles, in a majority-Hispanic school population. 63 staff members from a school in Chicago participated in a training initiative, followed by group consultation meetings over two years. Data were collected concerning educators’ progress in implementation of trauma-informed principles and barriers experienced. Improvements were found in five of SAMHSA’s principles, although no improvement was made in Cultural Humility. Heavy teacher workload, lack of family engagement, family stressors, and newcomer status were the most frequently-reported barriers. Findings support Safe First Steps as a promising framework for implementing trauma-informed care in schools serving minority youth. Results underscore the importance of support from families, school administrators, legislators, and community members.

## Introduction

Minority youth are disproportionately vulnerable to trauma due to a combination of psychosocial disparities and increased exposure to poverty, discrimination, community violence, and immigration stressors (Pumariega et al., 2022). Sacks and Murphey (2018) report that trauma exposure is particularly prevalent among minority youth, with 61% of Black non-Hispanic children and 51% of Hispanic children experiencing at least one potentially traumatic event. The nature of these experiences also vary across groups. Hispanic youth most commonly report exposure to financial hardship, separation from parents, and witnessing criminal activity, whereas Black youth are more likely to report exposure to community violence, household dysfunction, and parental divorce (Gordon et al., 2025).

The psychological effects of trauma also differ among racial and ethnic groups. Among Black youth, higher levels of trauma exposure are associated with increasing externalizing behaviors, while Hispanic youth tend to experience greater emotional, attention, and behavioral difficulties (Hunt et al., 2017). These effects extend beyond childhood, as trauma exposure can result in increased rates of depression, anxiety, post-traumatic stress, substance use disorders, and suicide (Pumariega et al., 2022). For youth of color who face barriers in accessing medical and mental healthcare, these impacts are exacerbated. Indeed, both Black and Hispanic individuals report mistrust of healthcare systems and stigma against mental health treatment due to experiences of discrimination (Fripp & Carlson, 2017). Other barriers also include limited resources to afford services, difficulty accessing treatment, and a lack of culturally-informed approaches in various treatment modalities (Snowden, 2003). Children may also have parents who do not speak English or do not understand the implications of trauma or the benefits of therapy (Snowden, 2003; Bailey et al., 2009).

However, schools may provide an alternative way to support trauma-affected minority youth. As schools are the only institution required by law to serve youth, they have the capacity to address the needs of children in under-resourced communities by building resources and being guided by trauma-informed principles (Goldenthal et al., 2024). In addition, schools are often viewed as a more trusted space, in which parents perceive their child’s learning environment to be filled with qualified individuals who are making decisions that are in the best interests of their child (Adams & Christenson, 2000; Bryk & Schneider, 2002). Therefore, families may be more likely to engage in services offered by a school as opposed to other institutions such as hospitals or outpatient treatment centers.

Dyer and Chisnell (2023) define trauma-informed care as identifying the protective and risk factors of trauma, comprehending the implications of trauma, and acknowledging the impacts of trauma on those who care for individuals who have experienced trauma. Although there has been an increase in school-based services in recent years, including the integration of social-emotional learning into class curricula, the absence of practices that are trauma-informed can cause students to experience retraumatization or unintentional triggering events (Maloney et al., 2024). Teachers of trauma-impacted children have also expressed feeling unprepared to help students due to limited awareness of trauma and desiring more support (Sun et al., 2024). Thus, given the disproportionate rate of trauma faced by minority youth, implementing trauma-informed practices in schools serving these populations may be able to equip teachers to better support students and contribute to positive student outcomes.

Indeed, prior literature has documented several examples of promising outcomes after implementing trauma-informed practices in minority-serving schools. For instance, school personnel became better equipped with the knowledge and tools needed to create safer, more supportive learning environments (NCTSN, 2021). Teachers have also reported improvements in students’ emotion regulation, academic competence, and classroom behavior after the implementation of trauma-informed practices within schools in Black communities (Mendelson et al., 2015). Among studies that have incorporated trauma-informed practices in schools serving Hispanic youth, outcomes have demonstrated lower teacher burnout and greater compassion satisfaction, as well as improvement in student self-efficacy (Christian-Brandt et al., 2020; Kataoka, 2018).

Yet, there is limited research on the practical implementation itself of trauma-informed programs themselves. In particular, Kisiel et al. (2024) maintains that there are inconsistencies in how trauma-informed components are implemented within schools of diverse youth populations. This is possibly due to the fact that components of trauma-informed care have been inconsistent across different definitions, and the extent to which trauma-informed principles have been implemented across various agencies varies (Hanson & Lang, 2016). In response to these challenges, the Safe First Steps initiative was developed to assess and support the implementation of trauma-informed practices within a public school serving predominantly Hispanic youth (University of Illinois Chicago, Urban Youth Trauma Center, 2021).

### Safe First Steps

The Safe First Steps training and consultation program is a two-year program designed to support providers in schools and other child-focused settings when working with families who have chronic trauma exposure (University of Illinois Chicago, Urban Youth Trauma Center, 2021). Safe First Steps draws upon Bronfenbrenner’s ecological theory, which emphasizes the interconnection between child development and the various individuals and contexts that children encounter, including schools (Bronfenbrenner, 1979). Grounded in the Substance Abuse and Mental Health Services Administration’s (SAMHSA) framework, this initiative follows six key principles: (1) Cultural, Historical, and Gender Issues, (2) Safety, (3) Trustworthiness and Transparency, (4) Collaboration and Mutuality, (5) Empowerment, Voice, and Choice, and (6) Peer Support (SAMHSA, 2014). By situating interventions within school contexts and equipping staff, Safe First Steps reflects models similar to other childhood consultation approaches that prioritize strengthening teachers to support student wellbeing (Cohen & Kaufman, 2000).

To align with SAMHSA’s recommendations for developing trauma-informed interventions through psychoeducation for teachers and staff (Chafouleas et al., 2016), the training component included a combination of lecture-style didactics led by trained facilitators and small group discussions. These groups were organized according to staff roles in the school and/or the grade levels they taught, following recommendations to provide targeted consultations for specific behavior plans and strategies (Shamblin et al., 2016). Over the course of a two-year partnership period, staff engaged in two additional training sessions and four group consultation meetings to reinforce the material and support practical implementation. Following SAMHSA’s theoretical framework, the content of the additional training focused on (1) implementation of restorative practices in school based settings, (2) strategies for increasing parental engagement in school-based settings, (3) the importance of self-care and managing secondary traumatic stress, and (4) trauma-informed approaches for addressing negative classroom behavior (SAMHSA, 2014). In addition to the training and consultations, Safe First Steps also provided therapeutic games, materials, and books focused on social-emotional learning that staff could use in classrooms during teaching activities and student free-time.

### Study Aims

The present study is a pilot evaluation of the Safe First Steps program, which aims to increase trauma awareness and bolster connections between school-based staff and other child service providers to support children, youth, and families who have been exposed to trauma or community violence. While evaluating student and teacher outcomes may aid in understanding the impact of trauma-informed practices in schools, research needs a greater focus on teachers’ competence in delivering trauma-informed care and the challenges that they face. By evaluating educators’ progress, research can identify areas for improvement in training and determine the type of support teachers need to address the most common challenges faced when working with trauma-impacted youth of color. The goal of the current study is to evaluate educators’ progress in trauma-informed care following a two-year training period and to identify the most commonly-cited challenges to implementation in a school serving majority-Hispanic youth.

## Methods

### Participants

This study included school-based service providers working with students in grades Pre-K to 8 that are affected by trauma or community violence. Due to the significant number of families who had reported past or ongoing exposure to trauma, an elementary school serving 872 students from Pre-K to Grade 8 (referred to as School A) located in northwest Chicago was identified as the pilot site for Safe First Steps and was included in the present study.

School personnel were recruited for the current study after participating in a Safe First Steps training. Following the completion of the training, participants willing to have their responses used for research purposes gave consent for their information to be included in this study. A total of 63 participants were included in the present study. Teachers or teaching assistants were 90.5% of the sample (*N* = 57), and the remaining participants were support staff (*N* = 2), mental health professionals (*N* = 2), and administrators (*N* = 2). 97% (*N* = 61) of participants held a Bachelor’s/Associate’s degree or higher. A full demographic breakdown of the participants, students in School A, and the broader community School A serves is available in Table 1.

**Table 1:** Demographic information

	Participant (Staff) Response (%) in School A	Student Response (%) in School A	Broader Community Response (%) in the (CMAP 2023) (CMAP 2023)
Hispanic	83%	84%	45%
Black	1%	5%	5%
White	13%	5%	32%
Asian	1%	5%	15%
Other	2%	1%	3%

Of the 63 participants initially enrolled, all participants completed both baseline and follow-up measures, resulting in no attrition over the two-year period. Although participation was voluntary, time was allocated during staff professional development time to complete study questionnaires and participants received automated reminders for data collection.

### Procedure

Participants signed up for the initial training through REDCap (Research Electronic Data Capture). Following the initial training session, participants completed the Baseline Safe First Steps Agency Trauma-Informed Practices Screening questionnaire. Participants then completed the Follow-Up Agency Trauma-Informed Practices Screening and the Implementation Plan Follow-Up form online via REDCap after the conclusion of the two-year partnership period.

### Measures

#### Safe First Steps Agency Trauma-Informed Practices Screening

This form was adapted from the New Orleans Trauma-Informed Schools Learning Collaborative’s Trauma-Informed Checklist (TISWTC 2017), which was used to evaluate the level of use of trauma-informed practices within organizations. The questionnaire includes six subscales based on the principles of trauma-informed care as defined by SAMHSA: (1) Cultural Humility, (2) Safety, (3) Trustworthiness and Transparency, (4) Collaboration and Mutuality, (5) Empowerment, Voice, and Choice, and (6) Peer Support. Each subscale contains four to six items scored between 0 (Never) and 3 (Always). Subscale scores were calculated by averaging the total scores of the items in each subscale. A total score was also calculated by averaging the subscale scores for each participant, with higher scores indicating greater implementation of trauma-informed care. The following scoring criteria was developed by the Safe First Steps team: an average score of 2.2 or higher would indicate strong performance or implementation (high); a score between 2.1 and 1.7 indicated an area of growth with active work toward implementation (medium); and a score of 1.6 or lower indicated a significant area for growth and limited implementation (low). Dividing scores into high, medium, and low categories was used to help inform what topics would be the focus of future training and consultation groups, targeting areas for changes in organizational practices.

1. *Cultural Humility*: The Cultural Humility subscale captures the extent to which staff move past stereotypes and biases based on identity while recognizing the unique cultural needs of individuals. Example items include, “Spaces are accessible and inviting to children and families” and “Families are permitted to express their culture and identity in multiple ways”.
2. *Safety*: The Safety subscale measures the extent to which staff and children feel physically and psychologically safe in physical settings and in interactions with one another (e.g. “Spaces are actively supervised by staff members”, “Staff maintain a calm demeanor when interacting with students”).
3. *Trustworthiness & Transparency*: The Trustworthiness and Transparency principle evaluates the transparency of decisions and operations within staff and between school personnel, children, and families (e.g. “Expectations for children are prominently displayed, communicated, and reviewed”, “Staff explore problems with children and families (i.e., what’s going on, what’s wrong) before discussing consequences”).
4. *Collaboration & Mutuality*: The Collaboration & Mutuality subscale describes the process of leveling the power differences among staff and recognizing the role that each person plays in trauma-informed care. Example items include, “Staff recognize and reward children’s strengths, interests, and hobbies” and “Children and families are encouraged to be active participants in their education, treatment, services provided, etc.”.
5. *Empowerment, Voice & Choice*: Empowerment, Voice, and Choice ensures that both staff and families build upon their strengths and feel in control (e.g. “Children are encouraged to develop emotional and behavioral self-awareness and regulation”, “Staff members actively and consistently seek out perspectives and opinions from children and families receiving services”).
6. *Peer Support:* The Peer Support subscale focuses on enhancing collaboration and building trust among those who have experienced trauma and those who are caring for individuals with traumatic experiences. Example items include, “Children are taught about how their behavior affects others, both positively and negatively” and “Staff members recognize one another’s personal strengths”.

#### Safe First Steps Implementation Plan Follow-Up

Based on the list of challenges shared in group discussions during the initial Safe First Steps training with School A, participants were asked to identify which of the following nine challenges they had experienced: (1) newcomer status, immigration issues, language barriers; (2) low family socioeconomic status, lack of resources; (3) difficulty with family engagement and parental involvement; (4) heavy workload, limited time to address student issues; (5) low academic performance, development deficits; (6) lack of trauma awareness and knowledge, absence of cultural knowledge; (7) increased family stressors, high student responsibility at home; (8) student engagement in risky behavior such as gang activity or substance use; and (9) difficulty in classroom with behavior management and emotion regulation. Participants were also asked, “To what degree do you feel that you have achieved your goals?” with responses ranging from “Not at all” (0) to “A lot” (3).

### Analytical Plan

All quantitative analyses were conducted using *IBM SPSS Statistics for Windows*,* Version 29.0* (IBM Corp., 2022). SPSS statistical software was used for all quantitative analysis. The present study used a within-subjects longitudinal design in which data were collected among the same groups of participants over a two-year period. Paired samples t-tests were conducted to assess changes in mean scores between baseline and follow-up across the six subscales and total score of the Trauma-Informed Practices Screening. Frequency analysis was conducted to identify the most commonly-cited challenges staff faced during implementation.

## Results

### Descriptive Statistics

Frequencies were calculated for the percentage of participants who had low, medium, and high scores in each subscale and in the total score of the Agency Trauma-Informed Practices Screening form, displayed in Table 2.

**Table 2:** Subscale scores across baseline and follow-up time points

Score	Cultural Humility		Safety		Trustworthiness and Transparency		Collaboration and Mutuality		Empowerment, Voice, and Choice		Peer Support		Total Score	
	Baseline	Follow-up	Baseline	Follow-up	Baseline	Follow-up	Baseline	Follow-up	Baseline	Follow- up	Baseline	Follow- up	Baseline	Follow- up
Low (%) [N]	22.2% [14]	4.8% [3]	6.3% [4]	3.2% [2]	20.6% [13]	11.1% [7]	6.3% [4]	0% [0]	6.3% [4]	4.8% [3]	9.5% [6]	1.6% [1]	4.8% [3]	0% [0]
Medium (%) [N]	31.7% [20]	25.4% [26]	44.4% [28]	25.1% [16]	36.5% [23]	20.6% [13]	44.4% [28]	17.5% [11]	52.4% [33]	27.0% [17]	34.9% [22]	20.6% [13]	39.7% [25]	7.9% [5]
High (%) [N]	54.0% [34]	69.8% [44]	49.2% [31]	71.4% [45]	42.9% [27]	68.3% [43]	49.2% [38]	82.5% [52]	41.3% [26]	68.3% [43]	55.6% [35]	77.8% [49]	55.6% [35]	92.1% [58]

As a pilot study, researchers wished to determine whether participants believed that they had successfully accomplished the goals for trauma-informed care that they created during the initial training. Responses to the question, “To what degree do you feel that you accomplished your goals?” in the Implementation Plan Follow-Up Assessment indicated that 63.5% of participants (*N* = 40) believe that they made a lot of the progress toward their goals, 30.2% of participants (*N* = 19) made “some” progress, and 6.3% of participants (*N* = 4) made “very little” progress. No participants responded that no progress had been made toward goal completion.

### Study Findings

The present study aimed to examine the implementation of trauma-informed practices in a school serving under-resourced youth of color. In addition, the study evaluated whether changes in implementation varied based on the number of challenges participants reported at the two-year follow-up. An a priori power analysis was conducted using *G*Power* (Faul et al., 2007) to determine the required sample size for detecting a medium effect (Cohen’s *d* = 0.50) with a significance level of α = 0.05 and power (1 – β) = 0.80. Results indicated that a minimum of 34 participants were required to detect a statistically significant difference using a paired samples t-test. Thus, the obtained sample size of *N* = 63 was sufficient for study aims.

Results from the paired samples t-test did not reveal statistically significant differences for the Cultural Humility subscale from baseline (*M* = 2.33, *SD* = 0.87) to follow-up (*M* = 2.41, *SD* = 0.46), *t*(62) = −0.70, *p* =.49. However, there were significant differences found between the Safety scores at baseline (*M* = 2.26, *SD* = 0.49) and follow-up (*M* = 2.52, *SD* = 0.44), *t*(62) = −5.10, *p* <.001. Significant differences were also found in the Trustworthiness and Transparency scores at baseline (*M* = 2.16, *SD* = 0.51) and at follow-up (*M* = 2.41, *SD* = 0.49), *t*(62) = −4.56, *p* <.001, and in the Collaboration and Mutuality scores at baseline (*M* = 2.25, *SD* = 0.48) and follow-up (*M* = 2.61, *SD* = 0.38), *t*(62) = −5.45, *p* <.001. Similarly, there were significant differences in Empowerment, Voice, and Choice subscale scores at baseline (*M* = 2.21, *SD* = 0.49) and follow-up (*M* = 2.47, *SD* = 0.61), *t*(62) = −4.02, *p* <.001, and in Peer Support scores at baseline (*M* = 2.36, *SD* = 0.63) and follow-up (*M* = 2.59, *SD* = 0.46), *t*(62) = −3.46, *p* <.001. Finally, total scores across the Agency Trauma-Informed Practices Screening also significantly differed between baseline (*M* = 2.23, *SD* = 0.47) and follow-up (*M* = 2.50, *SD* = 0.27), *t*(62) = −5.57, *p* <.001.

To evaluate the degree to which certain issues or stressors might interfere with the ability to improve implementation of trauma-informed practices over time, the current study assessed which challenges were experienced most frequently. The number of challenges selected by participants ranged from 2 to 9, and the average number of challenges reported was 4.44 (*SD* = 1.53). When examining the number of challenges experienced across the sample, results indicated that 49.2% of participants experienced 2–4 challenges (*N* = 31), and 50.8% of participants experienced five or more challenges (*N* = 32). The challenges most commonly reported included heavy workload and limited time to address student issues (*N* = 47), difficulty with family engagement and parental involvement (*N* = 43), increased family stressors or high student responsibility at home (*N* = 39), and newcomer status, immigration issues, or language barriers (*N* = 36).

## Discussion

The present study was a pilot evaluation of the Safe First Steps initiative, a program developed according to the six trauma-informed principles as defined by SAMHSA, and its implementation in a school serving majority-Hispanic youth. The implementation of Safe First Steps was associated with improvements in five of six trauma-informed principles: Safety, Trustworthiness and Transparency, Collaboration and Mutuality, Empowerment, Voice, and Choice, and Peer Support. No significant improvements were observed in Cultural Humility. The study also identified heavy workload, lack of family engagement, family stressors, and immigration-related issues as the top barriers to implementing trauma-informed principles.

Observed improvements in five of six principles of trauma-informed care may be linked with adherence to evidence-based recommendations. As per SAMHSA’s recommendations, Safe First Steps held ongoing and refresher training throughout the two-year implementation period (SAMHSA, 2014). Multiple, spread-out training sessions may have given staff the opportunity to practice their learning, receive support and feedback, and improve upon their knowledge (Stokes, 2022). The training sessions also focused specifically on barriers identified in previous research, including increasing parental involvement, decreasing teacher burnout, and establishing an implementation plan (Wassink-de Stigter et al., 2022; Kisiel et al., 2024), which may have contributed to staff-perceived improvement in implementation.

Lack of improvement in Cultural Humility may reflect systemic limitations rather than individual effort. Notably, items in the subscale measuring Cultural Humility are more focused on broad school climate (e.g. “Families are permitted to express their culture and identity in multiple ways”) in contrast to items in other subscales, which are more focused on direct staff behavior (e.g. ““Staff recognize and reward children’s strengths, interests, and hobbies”). Given that the majority of participants were teachers and teaching assistants (90.5%), it is possible that the subscale may not reflect individual educators’ efforts and progress in this principle, as the principle emphasizes institution-level responsiveness that may be out of participants’ control. Prior research has demonstrated that trauma-informed training was only effective for teachers when school administrators were fully supportive (Joyce & Showers, 2002). With the involvement of school leaders, procedural changes can be implemented school-wide, rather than solely in the classroom (Schimke et al., 2022). Future research should be intentional in recruiting school administrators and leaders as participants in trauma-informed training to evaluate how Cultural Humility may be improved at the school level with administrator involvement.

Commonly cited challenges in the present study mirror prior research. The most frequently cited challenge in this study was heavy workload. This is unsurprising in a setting that serves minority youth, as teachers serving children who disproportionately face trauma experience have higher rates of burnout and workload (Dorado et al., 2016). This finding adds to literature highlighting the need for more intentional self-care activities built into professional development as well as the involvement of administrators to make systemic changes to alleviate teacher responsibilities (State et al., 2024). Moreover, it is unsurprising for lack of family engagement and family stressors to be frequently cited challenges, given that 92.7% of families in School A were low-income. Many parents may work multiple jobs or have untraditional schedules, which can prevent engagement with school services (Miranda & Gonzales, 2022). To address this in future interventions, Ferlazzo (2011) recommends school-offered parent workshops to be held at a time accessible to parents and focused on a topic that addresses the needs of families. Finally, participants cited issues related to immigration as a significant barrier. Notably, School A experienced a large influx of new families during the two-year study period, many of which required support in domains outside school, including in housing and medical care. Schools can help newcomers by increasing funding for bilingual staff and additional mental health counselors to provide support, as well as providing resources that new families may need (Briggs et al., 2024). Together, these findings provide insight into barriers that schools serving majority-Hispanic students may experience.

The current study is strengthened by its longitudinal design and focus on underserved populations. However, there are notable limitations to take into consideration. Foremost, the evaluation of Safe First Steps in the present study did not have a control or comparison group, limiting the ability to attribute observed changes solely to the program itself. School A was selected as a pilot site based on high levels of trauma exposure among students and a demonstrated interest from school staff in adopting a trauma-informed framework. Given the ethical and practical considerations of withholding potentially beneficial training and support, a control group was not feasible at this stage. Instead, the focus of this pilot evaluation was to examine preliminary patterns of the Safe First Steps program in a naturalistic school setting. Thus, it is possible that other external factors such as district-level change may have influenced these findings. Moreover, the pilot only includes data collected from a single school. As a pilot study, the primary goal was to examine preliminary patterns before increasing the scale of Safe First Steps. Unfortunately, this limits the generalizability of the results to schools who do not share similar challenges or demographic characteristics. The school was selected for fitting the criteria of serving trauma-impacted youth of color from an underserved community, but future studies should collect data from multiple schools for more generalizability of results.

In addition, although the present study sought to provide trauma-informed training to all staff, the majority of study participants were teachers and teaching assistants. Other school roles in School A did not participate in the same professional development activities as teachers and often had conflicting schedules, so the majority of other school staff were unavailable for follow-up training and consultation group meetings. As a result, many of the challenges reported may have been skewed, as they were mostly challenges that teachers experienced. Including a greater diversity of school roles is needed in future studies to identify differences in challenges experienced between teachers and other school staff and responsibilities that various school roles may take on to contribute to the implementation of trauma-informed care.

Finally, data was collected only from school personnel and was not collected from students or parents. As a pilot evaluation, this study aimed to minimize participant burden and emphasized staff perspectives, given that staff directly participated in the Safe First Steps training and could provide insight into how trauma-informed practices were implemented within the school context. Future stages in evaluating Safe First Steps should expand data collection to include feedback from parents and students, as well as evaluating student outcomes.

## Conclusion

Given that youth of color are disproportionately exposed to trauma, there is a growing need for schools serving minoritized populations to adopt trauma-informed approaches. The present pilot study evaluated Safe First Steps as a training and consultation model designed to implement SAMHSA’s six trauma-informed care principles in a school serving a majority-Hispanic student population over a two-year period. Results demonstrated improvements in five of the six principles, with no statistically significant change observed in the Cultural Humility domain. Commonly reported barriers to implementation included heavy teacher workload, limited family engagement, cumulative family stressors, and challenges related to newcomer status.

While these findings provide preliminary support for Safe First Steps as a promising framework for implementing trauma-informed practices in schools serving diverse student populations, they should be interpreted with caution. The study’s lack of a control group, focus on a single school, and reliance on self-reported data from staff limit the study’s generalizability, internal validity, and capacity to support causal inferences. Future research should explore the program’s effectiveness across a broader range of school settings, incorporate comparison groups, and include perspectives from parents and students. Expanding the evaluation in this way would provide a more comprehensive understanding of how trauma-informed models can be sustainably and equitably implemented in educational settings.

## Data Availability

Data are available on request from the authors.
